# Assessing the Impact of Rotavirus Vaccination in Iranian Children Under Five: An Agent‐Based Modelling Approach

**DOI:** 10.1002/hsr2.72564

**Published:** 2026-05-25

**Authors:** Amirhesam Moosazadeh, Babak Eshrati, Ebrahim Babaee

**Affiliations:** ^1^ Vaccine Research Center Iran University of Medical Sciences Tehran Iran; ^2^ Preventive Medicine and Public Health Research Center, Psychosocial Health Research Institute, Department of Community and Family Medicine, School of Medicine Iran University of Medical Sciences Tehran Iran

**Keywords:** agent based model, contagious disease, epidemiological modeling, infectious disease, Rotasiil, Rotavirus vaccine

## Abstract

**Background and Aims:**

Rotavirus is a leading cause of severe diarrhoeal disease in young children worldwide. In 2024, Iran introduced the pentavalent oral vaccine Rotasiil® into its national immunization program. This study aimed to estimate its potential epidemiological impact using an agent‐based modeling (ABM) approach.

**Methods:**

An ABM was developed in NetLogo 6.4.0 to simulate rotavirus transmission among children under five in urban and rural settings. Agents represented individual children with attributes such as age, mobility, and susceptibility. Transmission occurred through contact within defined spatial grids, with higher probabilities assigned to rural settings to reflect poorer sanitation. Disease progression included incubation, symptomatic period, temporary immunity, and rare mortality. Two vaccination conditions (vaccinated vs. unvaccinated) were simulated, assuming 98% coverage and 55% seroconversion, consistent with Rotasiil‐Liquid trial data. Population turnover and births were incorporated. Each condition was run 1,000 times over one simulated year, and incidence rates and symptomatic days per child‐year were calculated. Sensitivity analyses tested low‐ and high‐incidence assumptions.

**Results:**

In urban settings, vaccination reduced incidence from 0.663 to 0.440 cases per child‐year (− 33.6%) and disease days from 3.895 to 2.584 (− 33.7%; *p* < 0.001). In rural populations, incidence declined from 0.670 to 0.488 (− 27.2%) and disease days from 3.968 to 2.886 (− 27.3%; *p* < 0.001). Sensitivity analyses showed consistent reductions of 26%–33% across incidence levels. Epidemic curves demonstrated lower peak intensity and more stable dynamics with vaccination.

**Conclusion:**

Rotasiil is expected to substantially reduce rotavirus burden in Iran, lowering incidence, symptomatic days, and epidemic peak size. These results support the inclusion of rotavirus vaccination as a key child health intervention, though continued post‐introduction surveillance remains essential.

## 1 Introduction

Diarrhoeal disease, characterised by passing at least three loose or watery stools within 24 h or by an increase in stool frequency beyond an individual's usual pattern, continues to represent a major health challenge worldwide. Each year, around 1.7 billion episodes are reported among children, placing it among the most widespread childhood illnesses. In 2021 alone, diarrhoeal disease accounted for around 9% of all global deaths in children under 5 years old—equating to more than 1,200 deaths every day or close to 444,000 over the year—despite the availability of simple and effective treatment options [1, 2].

Rotavirus, the predominant cause of severe diarrhoea among young children, is primarily transmitted via the faecal–oral route [3]. It is responsible for an estimated 35.2% (95% uncertainty interval: 28.7% to 43.0%) of diarrhoea‐related deaths in children under 5 years old. In 2021, it was linked to approximately 120,000 deaths from diarrhoea (ranging from 83,100 to 169,000) and contributed to 10.8 million disability‐adjusted life years (DALYs) globally (with a range of 7.52 to 15.2 million) [4].

Rotavirus gastroenteritis can manifest with a variety of symptoms, ranging from mild, short‐lived loose stools to severe watery diarrhea, often accompanied by vomiting, fever, abdominal pain, and lethargy. In severe cases, the condition can lead to rapid dehydration, electrolyte imbalances, and hypovolemic shock. Without prompt rehydration, these complications can be fatal, particularly in young children [3, 5]. No specific antiviral therapy exists for rotavirus. Treatment mainly focuses on supportive care, emphasizing the replacement of fluids and electrolytes, along with zinc supplementation. Dietary management, probiotics, antiemetics, and antisecretory agents may also be utilized [6, 7].

Countries that have implemented rotavirus vaccination programs report significant declines in rotavirus‐related illnesses and overall acute gastroenteritis among children, highlighting the vaccine's role in reducing the disease burden. The World Health Organization (WHO) recommends that all national immunization programs include live, oral, attenuated rotavirus vaccines. Four prequalified vaccines— Rotarix®, RotaTeq®, Rotavac®, and Rotasiil®—should be administered at 6, 10, and (for those on a 3‐dose schedule) 14 weeks of age alongside routine childhood vaccines. To minimize the risk of intussusception, the WHO advises starting the series before 15 weeks of age and completing it by 32 weeks of age. Clinical trials have demonstrated high efficacy in high‐income countries and moderate yet meaningful protection in low‐ and middle‐income settings [6, 8, 9].

Since late 2024, rotavirus vaccination has been incorporated into Iran's national immunization program using Rotasiil, a live, oral, pentavalent vaccine manufactured by the Serum Institute of India (SII). Rotasiil contains attenuated human‐bovine reassortant rotavirus strains that correspond to serotypes G1, G2, G3, G4, and G9, offering protection against severe rotavirus gastroenteritis. The vaccine complies with WHO quality and safety standards and has been approved by the Iranian Food and Drug Administration. In Iran, the formulation in use is the liquid two‐dose presentation (Rotasiil‐Liquid), supplied in ready‐to‐use 4 mL vials, each containing 2 mL of the dose. It is stored at 2°C–8°C, protected from light, and must not be frozen. Administration is recommended orally at ages 2, 4, and 6 months, with the first dose given before 15 weeks of age and all doses completed before 8 months. Rotasiil can be co‐administered with other vaccines, with oral vaccines administered prior to injectable ones. Under certain conditions, the interval between doses may be shortened to 1 month [10, 11].

Agent‐based modelling (ABM) is a computer simulation approach in which autonomous agents, each with defined attributes, behaviours, and interaction rules, operate within an environment to explore how individual actions affect system‐level outcomes [12, 13]. It can capture population heterogeneity, social network structures, stochastic variation, and emergent phenomena, making it well suited for modelling complex processes such as infectious disease transmission [14, 15].

Although rotavirus vaccination has only recently been incorporated into Iran's national immunization programme through the introduction of the pentavalent Rotasiil vaccine, the limited time since its adoption means that, to our knowledge, no peer‐reviewed studies have yet assessed its real‐world epidemiological impact in Iran using empirical surveillance data from hospital‐ or community‐based settings. To date, most Iranian research has focused on the economic feasibility of rotavirus vaccination. For instance, three previous modelling studies used decision‐analytic models, including a TRIVAC decision‐support model, a decision tree, and a Markov model, to estimate the cost‐effectiveness of introducing rotavirus vaccines in Iran [16, 17, 18]. All were based on earlier vaccine formulations (Rotarix and RotaTeq) rather than Rotasiil and relied primarily on aggregated epidemiological parameters, without explicitly capturing individual‐level transmission dynamics. More recently, a global modelling analysis that included Iran among 63 middle‐income countries provided cost‐effectiveness estimates for multiple products, including Rotasiil. Nevertheless, its primary focus was on cross‐country comparative economic evaluation, rather than a detailed, epidemiologically grounded, country‐specific assessment [19].

Given these evidence gaps, the present study applies an ABM approach to simulate rotavirus transmission and vaccination in the under‐five population of Iran, parameterised with nationally relevant epidemiological and demographic data. ABM enables explicit representation of individual‐level heterogeneity in susceptibility, contact patterns, and vaccine response, which are essential for capturing the complex dynamics of rotavirus spread and immunity. Using this framework, we assess the potential impact of Rotasiil introduction on rotavirus incidence and illness duration under Iranian conditions, thereby providing a context‐specific, mechanistic evaluation that complements previous economic analyses by focusing on key clinical components of disease burden rather than cost metrics. This approach aims to inform national decision‐makers by quantifying the expected improvements in these epidemiological indicators in the absence of direct post‐introduction surveillance data.

## 2 Methods

### 2.1 Modeling Approach and Software

**Figure 1:**
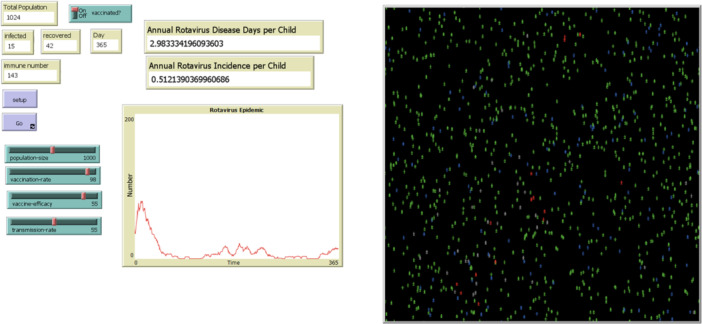
Urban model, one random run with vaccination in the NetLogo environment.

**Figure 2:**
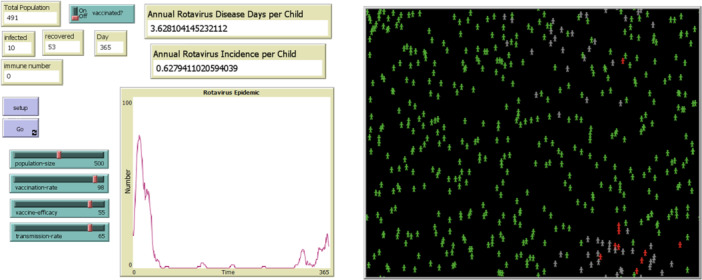
Rural model, one random run without vaccination in the NetLogo environment.

An agent‐based model was developed using NetLogo version 6.4.0, an open‐access simulation platform [20], to evaluate the effectiveness of the pentavalent live oral rotavirus vaccine Rotasiil in children under 5 years of age in Iran. Two distinct scenarios—urban and rural—were designed to account for differences in population size, density, and contact patterns. Each scenario was implemented as an independent model with specific initial conditions. The simulated environments in NetLogo, as generated in a single random run, are illustrated in Figures 1 and 2.

### 2.2 Agents and Environment

In this model, agents represent individual children aged between 0 and 5 years. At the beginning of each simulation run, each agent is assigned a random age on a continuous scale between 0 and 5 years. When agents reach the age of 5, they exit the model. The agents are randomly positioned on a grid at the start of each run.

The environment is structured as a grid of patches, with each patch representing a potential location for contact, such as homes, kindergartens, playgrounds, or parks. In the urban scenario, the environment consists of a 40 × 40 grid with 1,000 agents, while the rural scenario features a 25 × 20 grid with 500 agents. These population sizes reflect census data indicating that the number of children under 5 years old in Iran is approximately twice as high in urban areas compared to rural regions. It is important to note that the populations modeled do not represent the entire under‐five population of a city or rural area. Instead, they reflect a localized subset, such as children within one or several neighborhoods, where both direct and indirect contacts can occur.

### 2.3 Simulation Schedule and Movement Rules

For the simulation, one tick was assumed to represent 1 day, and each run lasted for a total of 365 ticks, equivalent to 1 year. Daily mobility levels varied between different settings to better reflect realistic conditions. In the urban model, children moved randomly each day by choosing a direction between 0 and 360 degrees and then moving two patches in that direction. In contrast, the rural model restricted daily movement to a single patch.

### 2.4 Disease Transmission and Epidemiological Parameters

Epidemiological parameters and assumptions are summarized in Table 1. At baseline, the point prevalence of diarrhea among children under five in Iran was estimated at 10 percent, based on a population‐based study that reported a prevalence of 10.3 percent [21]. As no community‐based estimate of the rotavirus‐attributable fraction was available, we applied a pooled value of 31 percent from an Iranian systematic review of outpatient gastroenteritis cases [22]. Multiplying these figures resulted in an initial rotavirus prevalence of 3.1 percent in the urban scenario (31 out of 1,000 individuals). In the rural scenario, a slightly higher prevalence of 3.4 percent was assigned (17 out of 500 individuals), reflecting evidence that the burden of diarrhea is generally greater in rural populations [23].

**Table 1:** Model parameters and assumptions for rural and urban settings.

Parameter	Urban model value	Rural model value	Description/source
Initial population	1000	500	Assumed based on scaled representation of rural and urban population sizes
Environment size	40 × 40 patches	25 × 20 patches	Adjusted according to relative population density in rural versus urban areas
Simulation duration	365 days (1 year)	365 days (1 year)	Fixed for all scenarios
Time step (tick)	1 day	1 day	Defined in NetLogo environment
Number of runs per scenario	1000	1000	To reduce stochastic variation and allow statistical analysis
Initial prevalence	3.1% (31 agents)	3.4% (17 agents)	Based on point prevalence of diarrhoea in Iran and proportion attributable to rotavirus
Transmission probability	55%	65%	[24, 25], Higher in rural areas due to closer household contact and reduced sanitation
Daily movement per agent	2 patches	1 patch	Reflects higher mobility in urban settings.
Incubation period	1–3 days	1–3 days	[26], Randomly assigned
Symptomatic period	5–7 days	5–7 days	[26], Randomly assigned
Temporary immunity duration	120 days	120 days	[27], no reinfection during this period
Disease‐specific mortality rate	0.0006	0.0006	[16], Per infection episode
Vaccination coverage	98%	98%	[28, 29], Based on national immunization program data
Vaccine efficacy (seroconversion)	55%	55%	[30], Based on clinical trial data for Rotasiil
Birth rate	1 every 30 days	1 every 60 days	[31], Approx. 13 per 1,000 annually

Transmission was modeled to occur whenever two or more individuals came within a distance of less than one patch during their daily movements. The probability of infection given such contact was set at 55 percent in the urban scenario [24, 25]. In the rural scenario, this probability was assumed to be 65 percent, representing a 10 percent relative increase to account for poorer sanitation and hygiene conditions, as well as more limited access to safe water.

The incubation period for rotavirus was randomly assigned to range from one to 3 days, while the symptomatic period was set between five and 7 days [26]. Temporary immunity following recovery lasted 120 days before individuals could become susceptible again [27]. Disease‐specific mortality was modeled at 0.0006 per infection episode, derived from an Iranian study [16] indicating that 3% of rotavirus cases are severe and that 2% of severe cases are fatal, resulting in an overall case fatality rate of 0.06%. Although mortality was incorporated into the model, the relatively small simulated populations (500 and 1,000 individuals) meant that deaths occurred infrequently. Therefore, the main outcomes of interest were incidence and illness duration rather than mortality.

### 2.5 Vaccination Scenarios

Two vaccination conditions were simulated for each setting. In the unvaccinated condition, no infant received the vaccine. In the vaccinated condition, all children younger than 1 year of age, whether present at the start of the simulation or entering later through births or turnover, were assigned a vaccination status probabilistically. Because rotavirus vaccination has only recently been incorporated into Iran's national immunization program and no reliable coverage data are yet available, a coverage rate of 98 percent was assumed, consistent with the uptake of other routine childhood vaccines [28, 29]. Among vaccinated infants, the probability of developing immunity was set at 55 percent, based on seroconversion rates reported in a clinical trial of Rotasiil‐Liquid [30], the formulation currently in use in Iran. Immunity was allocated immediately upon vaccination rather than by explicitly modeling the real‐world dosing schedule at 2, 4, and 6 months of age. An all‐or‐nothing assumption was applied, meaning that immune children were fully protected for the modeled duration, while non‐immune vaccinated children remained susceptible.

### 2.6 Population Dynamics

Population turnover occurred periodically. In the urban model, every 30 days, 65 new agents entered, two of whom were randomly infected at entry, while 45 exited the population. In addition, one newborn agent with age zero was introduced every 30 days to represent births [31]. In the rural model, every 60 days, 30 new agents entered, with one of them being randomly infected, while 15 agents exited. Similarly, one newborn agent with age zero was introduced every 60 days [31]. Entry position and age were randomly assigned for all new agents. In vaccination conditions, agents entering under 1 year of age followed the same probabilistic vaccination and seroconversion process described earlier.

### 2.7 Model Execution and Outcome Definitions

Each model condition was simulated over 1,000 independent stochastic runs. Considering both population settings (urban and rural), two vaccination scenarios (vaccinated and unvaccinated), and one baseline along with two alternative incidence assumptions examined in sensitivity analyses, a total of 12,000 runs were executed. This included 4,000 runs for the main analyses and 8,000 for sensitivity testing.

For each run, two primary epidemiological outcomes were calculated on a person‐time basis. The annual incidence rate of rotavirus was defined as the total number of new infection episodes divided by the total accumulated person‐time of the simulated population, expressed as cases per child‐year. Additionally, annual rotavirus disease days were defined as the total number of symptomatic person‐days summed across all children, divided by the total accumulated person‐time, expressed as disease days per child‐year. To illustrate infection dynamics, epidemic curves from randomly selected runs in each condition were plotted over the course of one simulated year.

### 2.8 Model Validation

Model validation involved both internal and face validity assessments. Internal validity was established by ensuring that the simulated baseline incidence was consistent with regional estimates. According to the WHO Eastern Mediterranean Region data, The average incidence of diarrhoeal disease was 2.1 episodes per child‐year (95% uncertainty interval 1.8–2.4) [32]. Multiplying this by the pooled Iranian rotavirus‐positive fraction of 31 percent [22] produced a target rotavirus incidence of 0.65 episodes per child‐year, which falls within a plausible range of 0.56–0.74. To align the model with this range, certain assumptions regarding environmental configuration (such as patch size and initial population distribution) were specified, while epidemiological parameters like transmission probability and disease duration were taken from published sources.

Face validity was evaluated through expert review. Three specialists independently assessed the plausibility of the model for simulating rotavirus transmission and vaccination in Iran. These included a specialist in epidemiological modeling, an epidemiologist, and a physician specialized in community medicine. They reviewed the assumptions concerning environment, agent behaviors, transmission dynamics, and epidemiological parameters, and confirmed that these were reasonable and appropriate for the study context.

### 2.9 Sensitivity Analysis

A sensitivity analysis was performed to evaluate the stability of results under different baseline incidence rates. The primary calibration aimed for a rotavirus incidence of between 0.56 and 0.74 episodes per child‐year. In addition, two other incidence levels were examined. One represented a lower incidence of approximately 0.4 episodes per child‐year, consistent with published evidence [33], and the other reflected a higher incidence exceeding 1.0 episodes per child‐year, as reported in another study [34]. To attain these incidence levels, the size of the simulated environment was adjusted while keeping all other model assumptions constant. This approach ensured representation of low, medium, and high incidence conditions, allowing for an evaluation of the robustness of model outcomes across a plausible epidemiological range.

### 2.10 Statistical Analysis

Simulation results were imported into Stata version 17 (StataCorp, College Station, TX, USA) for statistical processing. For each outcome measure in every model condition, means and 95 percent confidence intervals were calculated across the 1,000 runs. The distribution of results was assessed for normality, and two‐sample t‐tests were performed to evaluate differences between vaccinated and unvaccinated groups within each setting. A *p*‐value of less than 0.05 was considered statistically significant.

## 3 Results

### 3.1 Main Analysis

Across 1,000 stochastic simulations per condition, the mean estimated annual incidence per child‐year was higher in the unvaccinated groups than in the vaccinated groups, in both urban and rural populations of children under five.

In urban simulations, 1,000 independent runs indicated a mean incidence of 0.663 cases per child‐year without vaccination (95% CI: 0.657–0.669). With vaccination, the mean incidence dropped significantly to 0.440 (95% CI: 0.435–0.444), representing an absolute reduction of 0.223 cases per child‐year and a 33.6% decrease (*p* < 0.001).

In rural simulations of children under five, 1,000 independent runs yielded a mean incidence of 0.670 cases per child‐year without vaccination (95% CI: 0.662–0.677). With vaccination, incidence declined significantly to 0.488 (95% CI: 0.482–0.493), marking an absolute reduction of 0.182 cases per child‐year and a 27.2% decrease (*p* < 0.001). Although the rural group had a slightly higher baseline incidence than the urban group, vaccination still led to a substantial reduction in new infections.

Analysis of symptomatic illness demonstrated clear contrasts between conditions. In the urban population of children under five, those who did not receive vaccination experienced a mean of 3.895 disease days per child‐year (95% CI: 3.860–3.930). Among those who received vaccination, the mean number of disease days declined to 2.584 (95% CI: 2.560–2.609), a difference of 1.311 days corresponding to a reduction of roughly one‐third (*p* < 0.001).

The rural simulations indicated a slightly heavier baseline. Children under five without vaccination had a mean of 3.968 disease days per child‐year (95% CI: 3.925–4.010). In the vaccinated population, the average duration of illness decreased to 2.886 (95% CI: 2.853–2.919), equal to a decrease of 1.082 days, or just over one‐quarter of the total burden (*p* < 0.001). Thus, in both urban and rural settings, vaccination was associated with a lower mean number of symptomatic days in children under five. Mean annual incidence rates and average disease‐day estimates for each setting are shown in Table 2.

**Table 2:** Mean annual rotavirus incidence and disease days per child (95% CI) in urban and rural scenarios with and without vaccination, based on 1000 simulation runs and associated *p*‐values from *t*‐tests.

		Urban			Rural		
		Mean	95% CI	*p*. value	Mean	95% CI	*p*. value
Annual rotavirus incidenceper child							
	Without vaccination	0.663	0.657 to 0.669	< 0.001	0.670	0.662 to 0.677	< 0.001
	With vaccination	0.440	0.435 to 0.444		0.488	0.482 to 0.493	
Annual rotavirus disease days per child							
	Without vaccination	3.895	3.860 to 3.930	< 0.001	3.968	3.925 to 4.010	< 0.001
	With vaccination	2.584	2.560 to 2.609		2.886	2.853 to 2.919	

**Figure 3:**
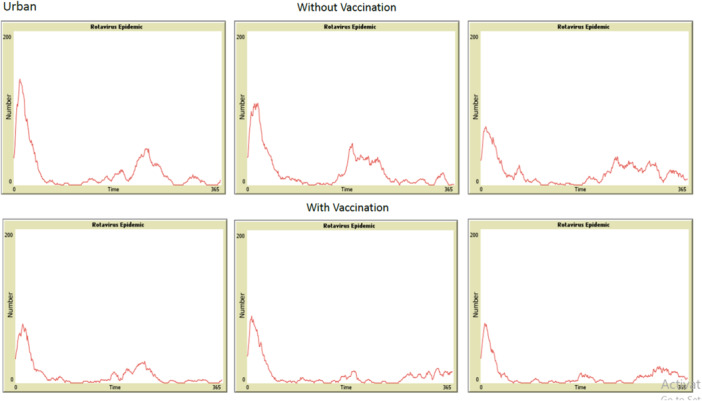
Epidemic curves from six independent random runs of the agent‐based model in the urban population under unvaccinated (top row) and vaccinated (bottom row) conditions.

**Figure 4:**
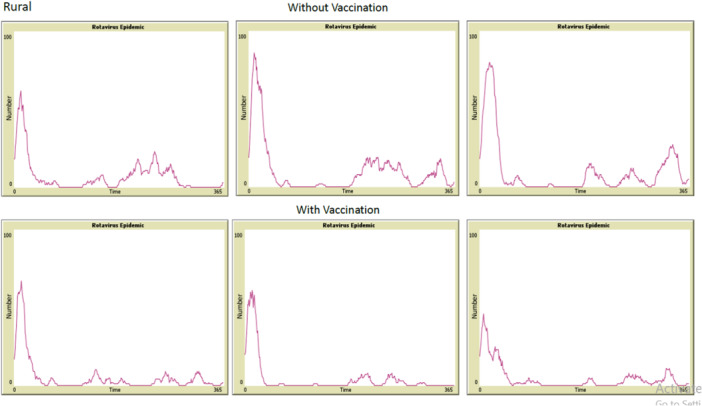
Epidemic curves from six independent random runs of the agent‐based model in the rural population under unvaccinated (top row) and vaccinated (bottom row) conditions.

Inspection of epidemic curves showed how vaccination shaped outbreak dynamics. Without vaccination, curves had tall, recurring peaks. After vaccination, the peaks were shorter and less pronounced (Figures 3 and 4). The horizontal axis shows time in days over one epidemiological year. The vertical axis shows the number of active infections at each time point. This steady drop in peak height shows that vaccination lowered both the average incidence and the intensity of epidemic waves over the simulated year.

### 3.2 Sensitivity Analysis

Under the low‐incidence condition, vaccination produced marked reductions in both the incidence rate and the average number of symptomatic days. In the urban setting, the mean annual incidence declined from 0.429 cases per child‐year (95% CI: 0.424–0.433) in the absence of vaccination to 0.305 (95% CI: 0.302–0.308) when vaccination was introduced, representing a 29% decrease (*p* < 0.001). Correspondingly, the mean number of disease days per child fell from 2.521 (95% CI: 2.495–2.547) to 1.792 (95% CI: 1.774–1.811), also a 29% reduction (*p* < 0.001). In rural populations, the average incidence rate declined by 26%, from 0.491 (95% CI: 0.486–0.497) to 0.363 (95% CI: 0.359–0.368) (*p* < 0.001). The mean number of illness days dropped in parallel, decreasing from 2.912 (95% CI: 2.879–2.944) to 2.148 (95% CI: 2.122–2.174), again a 26% reduction (*p* < 0.001).

In the high‐incidence condition, although the absolute incidence and illness‐day values were higher overall, the proportional effect of vaccination remained comparable. In urban simulations, incidence was reduced by 33%, falling from 1.014 cases per child‐year (95% CI: 1.001–1.021) without vaccination to 0.676 (95% CI: 0.671–0.682) with vaccination (*p* < 0.001). Average disease days in urban children dropped by the same proportion, from 5.964 (95% CI: 5.927–6.001) to 3.973 (95% CI: 3.940–4.001) (*p* < 0.001). In rural runs, incidence declined by 30%, from 1.120 (95% CI: 1.110–1.131) to 0.788 (95% CI: 0.780–0.796) (*p* < 0.001), with an identical 30% reduction in average disease days, from 6.637 (95% CI: 6.575–6.699) to 4.667 (95% CI: 4.621–4.716) (*p* < 0.001). Results of these sensitivity analyses are presented in Table 3.

**Table 3:** Sensitivity analysis of mean annual rotavirus incidence and disease days per child (95% CI) in urban and rural scenarios with and without vaccination, using Low‐ and High‐Incidence Estimates from Published Studies.

		Urban			Rural		
		Mean	95% CI	*p*. value	Mean	95% CI	*p*. value
**Low‐incidence setting**							
Annual rotavirus incidenceper child							
	Without vaccination	0.429	0.424 to 0.433	< 0.001	0.491	0.486 to 0.497	< 0.001
	With vaccination	0.305	0.302 to 0.308		0.363	0.359 to 0.368	
Annual rotavirus disease days per child							
	Without vaccination	2.521	2.495 to 2.547	< 0.001	2.912	2.879 to 2.944	< 0.001
	With vaccination	1.792	1.774 to 1.811		2.148	2.122 to 2.174	
**High‐incidence setting**							
Annual rotavirus incidenceper child							
	Without vaccination	1.014	1.001 to 1.021	< 0.001	1.120	1.110 to 1.131	< 0.001
	With vaccination	0.676	0.671 to 0.682		0.788	0.780 to 0.796	
Annual rotavirus disease days per child							
	Without vaccination	5.964	5.927 to 6.001	< 0.001	6.637	6.575 to 6.699	< 0.001
	With vaccination	3.973	3.940 to 4.001		4.667	4.621 to 4.716	

## 4 Discussion

Before the introduction of the first rotavirus vaccine in 2006, nearly all children worldwide were infected with rotavirus by the age of three to 5 years. The WHO prequalified RotaTeq in 2008 and Rotarix in 2009, followed by two additional products—Rotavac and Rotasiil—prequalified in 2018 [6]. By 2025, more than 130 countries had incorporated rotavirus vaccination into their national immunization programs, with worldwide vaccination coverage reaching approximately 59% [35]. In late 2024, Iran decided to introduce Rotasiil into its national immunization schedule, marking a significant milestone in diarrhoeal disease prevention. The benefits of vaccination are well established globally; analyses covering 69 countries included in the Global Rotavirus Surveillance Network (GRSN) demonstrated a 40% reduction in rotavirus prevalence after vaccine introduction [36].

This study applied an agent‐based modeling (ABM) framework to assess the potential epidemiological impact of rotavirus vaccination with Rotasiil among children under 5 years of age in Iran. By explicitly representing individual‐level heterogeneity, stochastic transmission dynamics, and spatial contact patterns, ABM enabled us to capture complex processes that are difficult to address using traditional compartmental models. Across 1,000 stochastic simulations per condition, vaccination consistently reduced both the incidence of rotavirus gastroenteritis and the average number of symptomatic illness days. In urban populations, the annual incidence rate declined by 33.6% (from 0.663 to 0.440 cases per child‐year), while in rural populations it fell by 27.2% (from 0.670 to 0.488). Symptomatic illness days showed comparable reductions, decreasing by 33.7% in urban settings and 27.3% in rural settings. Inspection of epidemic curves further demonstrated that vaccination not only reduced mean incidence but also flattened outbreak peaks, leading to a more stable epidemic profile over the simulated year.

Sensitivity analyses supported the consistency of these outcomes across different epidemiological contexts. Under low‐incidence conditions, vaccination reduced both incidence and symptomatic days by approximately 26%–29%. Under high‐incidence conditions, where the absolute burden was greater, vaccination still produced relative reductions of about 30%–33%. The consistency of the vaccine impact across low, medium, and high baseline incidence conditions suggests that the modeled effect is stable under diverse transmission scenarios, providing confidence that the projected benefits are not highly sensitive to baseline assumptions.

### 4.1 Comparison With Previous Studies

These findings align with modeling and observational studies showing that rotavirus vaccination offers substantial, though variable, benefits.

In India, a dynamic agent‐based model projected that nationwide rotavirus vaccination would reduce case prevalence by 33.7% (prediction interval: 30.7%–36.0%) [37]. The IndiaSim platform was also used to assess the health and economic impacts of introducing rotavirus vaccination, highlighting the utility of ABM for large, diverse populations [38]. While the IndiaSim analysis emphasized national mortality and financial outcomes, our study focused on incidence patterns, epidemic curves, and symptomatic illness days—objectives specifically relevant to Iran's epidemiological and policy context.

Alongside these modeling efforts, an observational study from India documented reductions in diarrheal disease after vaccination. The analysis found lower diarrhea prevalence in fully vaccinated children compared with their unvaccinated peers; specifically, there was a 16% reduction in the adjusted odds of diarrhea among those who received all three doses [39]. Although our projections and the Indian findings use different methods and populations, both consistently show that rotavirus vaccination reduces diarrheal disease. This consistency supports the intervention's effectiveness across contexts.

Post‐introduction surveillance from the Democratic Republic of the Congo supports these findings. A retrospective study across four hospitals in Kinshasa and Lubumbashi showed that, compared to the pre‐vaccine period, rotavirus positivity declined by 30.6% after Rotasiil introduction, with marked reductions in diarrhea, vomiting, and fever among children under five [40]. Likewise, in Kisangani, analyses showed that three‐dose vaccination reduced rotavirus prevalence among hospitalized diarrhea cases from 55.4% before to 23.1% after vaccine introduction, yielding an adjusted odds ratio of 0.31 (95% CI: 0.19–0.56) [41]. While these results are encouraging, they also show that rotavirus remains a significant contributor to pediatric gastroenteritis after vaccine introduction. This aligns with our model's prediction that vaccination substantially reduces, but does not eliminate, transmission in Iran.

Methodologically, our study differs from most empirical evaluations, which typically rely on observational surveillance and hospital‐based data. In contrast, by using an agent‐based modeling framework, we were able to capture demographic heterogeneity, stochastic transmission, and spatial contact patterns—factors often overlooked in real‐world studies. Notably, the consistency between our projections and empirical findings strengthens confidence that Rotasiil can substantially reduce the burden of rotavirus in diverse settings.

The epidemic curves in our model showed that vaccination, besides lowering average incidence, also reduced outbreak peak intensity. This pattern has important implications for the health system. In particular, smaller epidemic peaks mean less sudden pressure on hospitals, clinics, and inpatient capacity, helping prevent overcrowding and resource shortages during peak transmission [42]. Additionally, fewer symptomatic illness days among vaccinated populations result in more than just lowered incidence; this means improved quality of life for children and families, less parental caregiving or work absenteeism, and reduced medication and health service use [43]. Taken together, these findings suggest that vaccination benefits both individuals and the broader health system.

Although both our analysis and previous evaluations indicate that Rotasiil can meaningfully reduce rotavirus burden and that it has a favorable safety profile with no serious vaccine‐related adverse events reported [44], its use is not without challenges. Factors such as host and environmental conditions and the circulation of diverse rotavirus genotypes may influence real‐world effectiveness in Iran [45, 46]. These considerations highlight the importance of complementing modeled projections with robust post‐introduction surveillance, including monitoring of vaccine coverage, effectiveness, and strain distribution, to ensure that anticipated benefits are fully realized.

### 4.2 Strengths and Limitations

A key strength of this study is its ability to generate context‐specific evidence at a time when Iran has only recently introduced rotavirus vaccination into its national immunization program. The analysis distinguishes between urban and rural populations and tests alternative vaccination scenarios. This offers insights directly relevant for policymakers during the early implementation phase. Another advantage is the emphasis on Rotasiil, the vaccine currently adopted in Iran. This focus makes the findings directly applicable to the national context and distinguishes this work from previous modeling efforts that primarily examined Rotarix or RotaTeq.

Using agent‐based modeling provided a flexible platform for simulating complex transmission processes under real‐world conditions. This framework is relevant for policy now and can be adapted for future evaluations of immunization strategies or new vaccine introductions. Together, these strengths highlight the practical and forward‐looking contributions of the study to rotavirus control in Iran.

The findings of this study should be interpreted in light of several limitations. Certain structural and contextual factors could not be explicitly simulated in this framework due to the absence of detailed empirical data. For example, precise differences in urban versus rural population density, contact networks, and movement patterns were simplified in the model design.

Overall, the analysis relied on available data and a series of assumptions. Although these were selected from the best available sources, they inevitably introduce some uncertainty. To address this, sensitivity analyses were performed across different incidence levels. This helped confirm that the main conclusions were not dependent on a single baseline estimate.

In modelling vaccination, immunity was implemented in an “all‐or‐nothing” fashion at the start of the simulation. This approach did not reflect the real three‐dose schedule or the short‐term delay to seroconversion after each dose. The simplification reduced complexity and allowed focus on annual outcomes, but it may have influenced short‐term dynamics and seasonal timing of protection.

Seasonal variation in rotavirus incidence was not captured because detailed community‐based surveillance data are lacking in Iran. Excluding this factor limited the model's ability to reproduce temporal fluctuations in disease transmission.

For clarity and feasibility, certain other determinants of real‐world vaccine performance were held constant. These included genotype diversity, potential waning of immunity, and broader environmental or health system changes, such as sanitation and healthcare access. These exclusions allowed a transparent assessment of vaccination impact, but they highlight areas for refinement as more data become available.

This study focused mainly on incidence and symptomatic days. It did not explicitly model mortality, hospitalization, or economic burden. Although reducing incidence is expected to proportionally lower these outcomes, detailed evaluation of mortality, hospitalization, and economic effects remains a key opportunity for future research, which may further clarify the full impact of such interventions.

## 5 Conclusion

This study shows that introducing Rotasiil into Iran's national immunization program can substantially reduce rotavirus gastroenteritis among children under five. Using an agent‐based modeling framework, we found consistent reductions in incidence, symptomatic illness days, and epidemic peak intensity across both urban and rural populations and under diverse incidence scenarios. These results align with global evidence, supporting the effectiveness of rotavirus vaccination in reducing disease burden and alleviating pressure on health systems.

However, as with any modeling study, the findings should not be interpreted as definitive predictions. The model involved simplifying assumptions and relied on available data, meaning real‐world outcomes may vary depending on vaccine coverage, circulating strains, and health system factors. Continued post‐introduction surveillance and context‐specific evaluations remain essential to fully capture the impact of vaccination in Iran. Sustained efforts in monitoring and adaptation will help maximize the long‐term benefits for children's health nationwide.

Rotasiil is expected to substantially reduce rotavirus burden in Iran, lowering incidence, symptomatic days, and epidemic peak size. These results support the inclusion of rotavirus vaccination as a key child health intervention, though continued post‐introduction surveillance remains essential to assess the impact of vaccination by using data from real‐word conditions of vaccine use.

## Author Contributions

**Amirhesam Moosazadeh:** conceptualization, methodology, visualization, writing – original draft, software, formal analysis, project administration, data curation. **Babak Eshrati:** supervision, conceptualization, methodology, software, writing – review and editing, validation. **Ebrahim Babaee:** conceptualization, supervision, software, formal analysis, validation, methodology, writing – review and editing, data curation.

## Funding

The authors have nothing to report.

## Ethics Statement

Ethical approval for this study was obtained from the Research Ethics Committee of Iran University of Medical Sciences (Approval Code: IR.IUMS. REC.1403.508). The analysis relied exclusively on secondary data from published sources, and no individual‐level or identifiable personal data were used.

## Conflicts of Interest

The authors declare no conflicts of interest.

## Transparency Statement

The lead author Ebrahim Babaee affirms that this manuscript is an honest, accurate, and transparent account of the study being reported; that no important aspects of the study have been omitted; and that any discrepancies from the study as planned (and, if relevant, registered) have been explained.

## Data Availability

The data supporting the findings of this study are available from the corresponding author upon reasonable request.
